# Evaluation of eating habits and lifestyle in patients with obesity before and after bariatric surgery: a single Italian center experience

**DOI:** 10.1186/s40064-016-3133-1

**Published:** 2016-09-01

**Authors:** Hellas Cena, Rachele De Giuseppe, Ginevra Biino, Francesca Persico, Ambra Ciliberto, Alessandro Giovanelli, Fatima Cody Stanford

**Affiliations:** 1 Unit of Human Nutrition and Dietetics, Department of Public Health, Experimental and Forensic Medicine, University of Pavia, via Bassi 21, 27100 Pavia, PV Italy; 2 Scuola di Specializzazione in Scienza dell’Alimentazione, University of Milan, via GB Grassi 74, 20157 Milan, Italy; 3 Institute of Molecular Genetics, National Research Council of Italy, via Abbiategrasso 207, 27100 Pavia, Italy; 4 Department of General Surgery, Istituto Clinico S. Ambrogio, via Faravelli 16, 20149 Milan, Italy; 5 Department of Medicine and Pediatrics, Massachusetts General Hospital, Harvard Medical School, Boston, MA 02114 USA

**Keywords:** Gastric bypass roux-en-Y, Sleeve gastrectomy, Physical activity, Dietary habits, Smoking habits, Bariatric surgery, Weight loss

## Abstract

**Background:**

The study evaluated and compared the eating habits and lifestyle of patients with moderate to severe obesity who have undergone Roux-en-Y Gastric Bypass (RYGB) and Sleeve Gastrectomy (SG).

**Methods:**

Food frequency (FF), food habits (FH), physical activity and life style (PA) as well as smoking habits (SH) were analyzed in 50 RYGB (25 M; aged: 24–64) and 50 SG patients (25 M; aged: 22–63) by means of a validated questionnaire, before (T_0_) and 6 months (T_1_) post bariatric surgery. A score for each section (FF, FH, PA, SH) was calculated.

**Results:**

ANOVA analysis (age/sex adjusted): FF and FH scores improved at T_1_ (RYGB and SG: *p* < 0.001); PA score improved but not significantly; SH score did not change at T_1_ neither in RYGB nor in SG. Mixed models: FF and PA scores did not correlate with age, gender, weight, BMI, neither in RYGB nor in SG; FH score was negatively correlated both with weight (RYGB: *p* = 0.002) and BMI (SG: *p* = 0.003); SH score was positively correlated with age, in SG (*p* = 0.002); the correlation was stronger in females than in males (*p* = 0.004).

**Conclusions:**

Although dietary habits improved, patients did not change their physical activity level or their smoking habits. Patients should receive adequate lifestyle counseling to ensure the maximal benefit from bariatric surgery.

## Background

The rate of obesity has more than doubled over the past 30 years in most countries of northern Europe including the UK and Scandinavian countries, as well as in many southern European countries. In the past three decades, childhood overweight and obesity prevalence has risen substantially in most high-income countries; moreover it seems to be rising rapidly in low-income and middle-income countries (Lobstein et al. 2015). The prevalence of obesity in the European adult population is about 15.5 %. Among European countries, Italy has the lowest adult obesity prevalence at approximately 9 % (8.5 % men, 9.4 % women) (Santonicola et al. 2013). Yet, in contrast, it has the highest rate of childhood obesity (Ahrens et al. 2014; The OECD Report Obesity and the Economics of Prevention: Fit not Fat 2014).

When conservative strategies such as diet improvement, behavior modifications, and physical activity do not produce effective weight loss outcomes, bariatric surgery is considered the most effective treatment for moderate to severe obesity in order to produce significant and sustained weight loss and to markedly reduce obesity co-morbidities (Livingston 2007).

However, it has been reported that there is a high incidence of weight regain from the nadir weight in the second year following surgery (ranging from 46 to 63 % regain of weight initially lost at the nadir) (Freire et al. 2012) with a significant relapse in obesity co-morbidities and, consequently, a deterioration in the patient’s quality of life. Inadequate weight loss or weight regain after bariatric surgery, along with micronutrient deficiencies, are longitudinal risks that have to be monitored in the bariatric surgery patient population. (Concors et al. 2016). Inadequate weight loss or weight regain may be secondary to a myriad of factors including pre-operative high body mass index (BMI), concomitant psychological disorders, gastric pouch and gastrojejunal anastomosis dilation (Iannelli et al. 2013) and many other post-surgical complications related to the anatomic alteration (Concors et al. 2016; Pandolfino et al. 2004) but also to the lack of improvement in eating habits and a persistent sedentary lifestyle which might lead to unsuccessful maintenance of body weight reduction (Soares et al. 2014). Freire et al. (2012) reported that poor diet quality, lack of physical exercise, and poor nutritional counseling follow-up visits were significant positive predictors of weight regain in patients after bariatric surgery. Concomitantly, Papalaszarou et al. (2010) reported that weight loss varied considerably even among patients undergoing the same surgical procedure depending on eating behavior modifications, food consumption frequency, and physical activity which may affect weight changes postoperatively. For these reasons, it is important for bariatric patients to be monitored and counseled through interviews and questionnaires in the pre-operative period and over the duration of their life to ensure proper nutrition and behaviors to support their bariatric surgery outcomes.

Physical activity, regardless of weight loss, provides numerous health benefits especially for individuals who are overweight and obese as they are at risk for metabolic and/or cardiovascular diseases (Swift et al. 2014).

Several studies show that physical activity presents several benefits in individuals with obesity: improvement of co-morbidities, mortality, and quality of life (Flancbaum et al. 2006), and physical activity is important for weight maintenance (Thomas et al. 2015). PA plays a major role in minimizing the amount of weight regained after initial weight loss. (Swift et al. 2014). Patients with obesity should be encouraged to adhere to PA programs over the long-term regardless of the amount of weight loss achieved, since metabolic and cardiovascular benefits may be achieved even in the absence of weight loss (Swift et al. 2014).

A recent meta-analysis showed that although bariatric surgery is more effective than lifestyle interventions for the treatment of severe obesity and its co-morbidities, some individuals have a striking response to lifestyle interventions and bariatric surgery is insufficient to treat all patients with severe obesity (Baillot et al. 2015). Previous research has shown that many bariatric surgery patients have low physical activity levels and spend 80 % of their time in sedentary behaviors (Bond et al. 2012).

Several studies demonstrate that well-maintained weight loss outcomes after bariatric surgery may be achieved with a consistent postoperative physical exercise program (Gradaschi et al. 2014; Egberts et al. 2011).

Previous knowledge reports an inverse relationship between BMI and smoking habits in individuals with normal weight (Lin et al. 2004; Robb et al. 2008; Pisinger and Jorgensen 2007); however, it has been demonstrated that in patients with overweight and obesity, there is a positive correlation with BMI (Chatkin et al. 2010) and that smoking leads to the development of insulin resistance and metabolic syndrome (Cena et al. 2011) with higher waist circumference as well as BMI in heavy smokers. (Cena et al. 2013).

There are reasons why there may be a correlation between smoking habits and obesity. People with obesity often smoke more as they feel it will help with weight loss (Fulkerson and French 2003). It is also possible that higher stress levels as well as frequent psychological discomfort could contribute to an ongoing smoking habit (Chatkin et al. 2010). The Italian Guidelines for bariatric surgery (Forestieri 2015) state that preoperative weight loss and smoking cessation decrease operative mortality, but patients are allowed to proceed with bariatric surgery if they are smokers.

The increased perioperative morbidity related with cigarette smoking is thought to be a combination of both its long-term health consequences and acute toxic effects (Haskins et al. 2014).

A recent study conducted by Hanskins et al. (2014) on 41,445 patients undergoing bariatric surgery (35,696 laparoscopic; 5749 open), revealed that smoking significantly increased pulmonary complications (prolonged intubation, re-intubation, and pneumonia), organ space infection, and length of hospital stay in all types of bariatric surgery. Therefore, smoking cessation is encouraged in order to minimize postoperative morbidity in bariatric surgery.

Based on these lifestyle considerations, the aim of the present study was to evaluate and compare dietary habits and lifestyle patterns, especially physical activity and smoking habits, of patients with obesity who have undergone Roux-en-Y Gastric Bypass (RYGB) and Sleeve Gastrectomy (SG) by using a validated self-administered questionnaire (Turconi et al. 2003) to ascertain the adequacy of lifestyle changes.

## Methods

### Subjects

The present cohort study evaluated 50 consecutive (i.e. in the order of appearance to our clinic) patients who underwent Gastric Bypass Roux-en-Y (RYGB; 25 M/25F) and 50 consecutive patients who underwent Sleeve Gastrectomy (SG; 25M/25F), according to the S.I.C.OB. Guidelines (Forestieri 2015) and attending the Department of General Surgery, Istituto Clinico S. Ambrogio, Milano, Italy between 2013 and 2014.

The patients were enrolled to ensure an equal number of males and females for both operations. All patients compile a self-administered structured dietary questionnaire (Turconi et al. 2003) before (T_0_) and 6 months post bariatric surgery procedures (T_1_) as part of their standard pre-op assessment and follow-up visits as part of the standard hospital protocol, data collection included anthropometric measurements (height, weight, BMI,) both at T_0_ and at T_1_.

After bariatric surgery dietetic counselling and education in nutrition was provided by trained professionals (dietitians and health coaches) at every visit, promoting physical activity to achieve lifestyle changes and improve long-term results, according to the S.I.C.OB. Guidelines (Forestieri 2015).

Informed consent was obtained from all individual participants included in the study.

### Anthropometric parameters and body composition

Weight and height were measured according to standard conditions. Body weight was measured with subjects wearing only their underwear and without shoes by means of a steelyard scale (precision ± 100 g); body height was measured on subjects without shoes by means of a stadiometer (precision ± 1 mm). BMI was then calculated as a ratio between weight and height squared with weight in kilograms and height in meters.

### Dietary and lifestyle questionnaire

One of the most commonly used methods to evaluate nutritional quality is the semi-quantitative food frequency questionnaires (FFQs) as they are brief, inexpensive, and easy to administer. Nevertheless, FFQs are designed to measure energy consumption and dietary intake of macro and micronutrients, but they do not provide information on other important aspects bariatric surgery patients such as their food habits, eating behaviors, and physical activity patterns, which are important to address in any nutritional surveillance program. Therefore, other questionnaires, structured with scores and scale scores, which aim to investigate some of the above-mentioned deficiencies, have been developed (Turconi et al. 2003).

Out of nine sections of a previously validated self-administered dietary questionnaire (Turconi et al. 2003), we evaluated three sections aimed at investigating food frequencies, dietary habits, and physical activity patterns. Of note, the questionnaire was drawn from one originally developed and validated on an Italian youth population (Turconi et al. 2003) and then adapted by two dietitians to our adult population before its administration. The new adapted version was previously piloted on a sample of 24 subjects and revised accordingly, although its validity and reliability were not formally tested. The study subjects were trained on how to complete the questionnaire (i.e. each subject received 15 min of instruction by the two dietitians) to ensure accuracy.

The Food Frequency section (FF), assessed daily consumption of typical food and beverages such as bread, pasta, cereal products, fruit and vegetables, milk, tea, coffee and weekly consumption of other foods such as meat and meat products, fish, eggs, cheese, legumes, etc. Alcoholic beverage intake was also investigated (Turconi et al. 2003).

The Food Habit section (FH), was designed to investigate the food habits related to breakfast consumption, daily number of meals, and consumption of fruit, vegetables, and soft drinks or alcoholic beverages (Turconi et al. 2003).

Each section consisted of questions with the following response categories: always, often, sometimes, never. The score assigned to each response ranged from 0 to 3, with the maximum score assigned to the healthiest one and the minimum score to the least healthy one according to the National Dietary Guidelines (INRAN 2015). Each section of the questionnaire was scored. The total score was divided into tertiles, where the lowest one referred to “inadequate eating habits”, the medium one referred to “partially satisfactory eating habits” and the highest one referred to “satisfactory eating habits”, according to the National Dietary Guidelines (INRAN 2015).

The Physical Activity and Life Style section (PA) investigated lifestyle and physical activity levels. All answers were structured to quantify the time spent weekly in physical activity, to investigate the activities spent during the free time (such as walking, watching TV, listening, to music, using the computer, reading a book, practicing a sport and shopping), and to quantify the hours spent daily on the computer or in watching TV (Turconi et al. 2003); each score ranging from 0 to 3, with the maximum score assigned to the healthiest habit. The total score was divided into tertiles, where the lowest one referred to “sedentary physical level”, the medium one referred to “partially moderate physical level” and the highest one referred to “active physical level” according to the National Lifestyle Guidelines (Linee Guida per un Corretto Stile di Vita 2015).

Separately, patients were classified as current, never, or former smokers, and their smoking habits were also investigated with six semi-quantitative questions such as:

- “Are you smoker, no-smoker or former smoker?”;
- “How long have you been smoking?”, “How old were you when you started smoking?” and “How many cigarettes do you smoke daily?” in current smokers;
- “How many years have passed since the last cigarette?” and “How many cigarettes per day did you use to smoke?” in former smokers.

Each question included multiple choices coded from a score ranging from 1 to 3. Overall, the *minimum* score was assigned to the healthiest habit.

### Statistical analysis

Data quality control and statistical analyses were performed using STATA 11 (StataCorp, College Station, TX). Descriptive statistics representing means, percentages, standard deviations, tertiles, ranges and 95 % confidence intervals (CI) were computed. T test was performed to verify that patients’ characteristics did not differ significantly in the two surgery groups at baseline. Repeated measures ANOVA were used to compare questionnaire scores before and after surgery taking into account age and sex. Mixed effect linear regression analysis was performed to evaluate if any of the collected variables were related with questionnaire scores, where the random part of the model takes into account the correlation within subjects.

## Results

### Results at baseline T_0_

As shown in Table 1, where baseline patients’ characteristics and anthropometric measurements are reported, RYGB and SG groups did not differ significantly for age, height, weight, and BMI.

**Table 1:** Baseline patients’ characteristics and anthropometric measurements

	RYGB *n* = *50* (*25F*/*25M*)		SG *n* = *50* (*25F*/*25M*)		
	Mean ± SD	Range	Mean ± SD	Range	*p*
Age (years)	42.7 ± 10	24–64	40.4 ± 10.5	22–63	0.26
Height (m)	1.7 ± 0.1	1.54–1.84	1.7 ± 0.1	1.5–1.86	0.49
Weight (kg)	129.8 ± 22.7	94–199	127.8 ± 28.8	86–206	0.69
BMI (kg/m^2^)	44.8 ± 6.8	35.3–65	44.5 ± 7.4	32.1–62.9	0.84
Smoking habit (score)	3.7 ± 4.4	0.0–12.0	4.6 ± 4.9	0.0–12.0	0.36

Data are reported as mean ± standard deviation (SD) and *range*

*p* value refers to t-Student Test for unpaired data

Smoking habit was investigated with six semi-quantitative questions. Each question included multiple choices coded from a score ranging from 1 to 3; the minimum score was assigned to the healthiest habit

*BMI* body mass index

All patients completed the questionnaires except two SG males, then each section of the questionnaire was scored and these scores were placed into tertiles in order to describe eating behaviors and physical activity levels at baseline: the worst evaluation was assigned to the lowest tertile while the best evaluation was assigned to the highest (Table 2).

**Table 2:** Percentage distribution of subjects according to Scores’ Tertiles

Surgical techniques (*n* = *sample size*)	1st tertile	2nd tertile	3rd tertile
	n (%)	n (%)	n (%)
RYGB *n* = *50* (*25F*/*25M*)			
FF—food frequency	22 (44)	19 (38)	9 (18)
FH—food habits	20 (40)	19 (38)	11 (22)
PA—physical activity and lifestyle	24 (48)	16 (32)	10 (20)
SG *n* = *50* (*25F*/*25M*)			
FF—food frequency	13 (27.08)	23 (47.92)	12 (25)
FH—food habits	16 (33.33)	18 (37.5)	14 (29.17)
PA—physical activity and lifestyle	11 (22.92)	19 (39.58)	18 (37.5)
Overall *n* = *100* (*50F*/*50M*)			
FF—food frequency	35 (35.71)	42 (42.86)	21 (21.43)
FH—food habits	36 (36.73)	37 (37.76)	25 (25.51)
PA—physical activity and lifestyle	35 (35.71)	35 (35.71)	28 (28.57)

Values defining tertile intervals are respectively: 21–31, 32–36 and, 37–47 for questionnaire section FF; 14–23, 24–27 and, 28–34 for section FH; 0–3, 4–5 and, 6–12 for section PA

The worst evaluation was assigned to the lowest tertile while the best evaluation was assigned to the highest

*RYGB* Roux-en-Y gastric bypass, *SG sleeve gastrectomy*

Considering smoking habit at T_0_ in RYGB population, 30 % of patients (n = 15) were current, 18 % (n = 9) were former and 52 % (n = 26) were never smokers while in SG population, 39.6 % of patients (n = 19) were current, 12.5 % (n = 6) were former and 47.9 % (n = 23) were never smokers.

At baseline, there was no statistically significant difference of questionnaire scores between the two surgery groups with an exception of the section PA- Physical Activity and Lifestyle (age and sex-adjusted values: 3.5, 95 % CI 2.8–4.1 in RYGB, 5.3, 95 % CI 4.6–5.9 in SG).

Additionally, we reported a negative correlation between FH-section score and weight at T_0_ (r = −0.32; *p* = 0.024), only in RYGB patients.

### Results of food frequency (FF) and food habits (FH) section at T_1_

Repeated measures ANOVA showed that FF-and FH- section scores improved significantly both in RYGB (*p* = 0.0017 and *p* < 0.0001, respectively) and in SG (*p* = 0.0002 and *p* < 0.0001, respectively), after bariatric surgery, when adjusted for sex and age (Table 3).

**Table 3:** Results of repeated measures ANOVA on questionnaire scores by surgery

	T_0_	T_1_	Sex^a^	Age^a^	p^b^	Wilks’ Λ
	Mean ± SD	Mean ± SD				
RYGB *n* = *50* (*25F*/*25M*)						
FF—food frequency	32.9 (4.9)	39.5 (3.3)	0.5744	0.5219	0.0017	0.809
FH—food habits	24.5 (4.3)	34.4 (2.6)	0.0162	0.0781	<0.0001	0.6327
PA—physical activity and lifestyle	3.6 (2.4)	6.1 (3.1)	0.0501	0.6788	0.4431	0.9874
Smoking habit	3.7 (4.4)	3.7 (4.4)	*n.a.*	*n.a.*	*n.a.*	*n.a.*
SG *n* = *48* (*25F*/*23F*)						
FF—food frequency	33.8 (4)	38.2 (3.2)	0.6475	0.1116	0.0002	0.7266
FH—food habits	24.7 (4.8)	33.5 (2.6)	0.0032	0.4936	<0.0001	0.6348
PA—physical activity and lifestyle	5.2 (2.3)	7.9 (3.4)	0.0199	0.639	0.2603	0.9719
Smoking habit	4.6 (4.9)	4.6 (4.9)	*n.a.*	*n.a.*	*n.a.*	*n.a.*

T_0_ and T_1_ scores are reported as mean ± standard deviation (SD)

FF, FH and PA scores were placed into tertiles; the worst evaluation was assigned to the lowest tertile while the best evaluation was assigned to the highest

Smoking habit was investigated with six semi-quantitative questions. Each question included multiple choices coded from a score ranging from 1 to 3; the minimum score was assigned to the healthiest habit

*n.a.* not applicable, *RYGB* Roux-en-Y Gastric Bypass, *SG* sleeve gastrectomy

^a^ *p* value relative to the significance of the term in the analysis

^b^Age- and sex-adjusted *p* value relative to testing equality of scores before and after the surgery

On average section FF and FH, questionnaire scores improved more in subjects who underwent RYGB surgery, even without reaching statistical significance (mean score delta 6.6 vs. 4.4 for FF- score; mean score delta 9.9 vs. 8.8 for FH-score; respectively).

Mixed models by surgery reported that FF section score was not correlated with age, gender, weight, or BMI, either in RYGB patients or in SG patients; however FH section score was negatively correlated both with weight (β = −0.34; *p* = 0.002) and with BMI (β = −0.15; *p* = 0.003) respectively in RYGB and SG.

### Results of physical activity and lifestyle (PA) section at T_1_

**Fig. 1:**
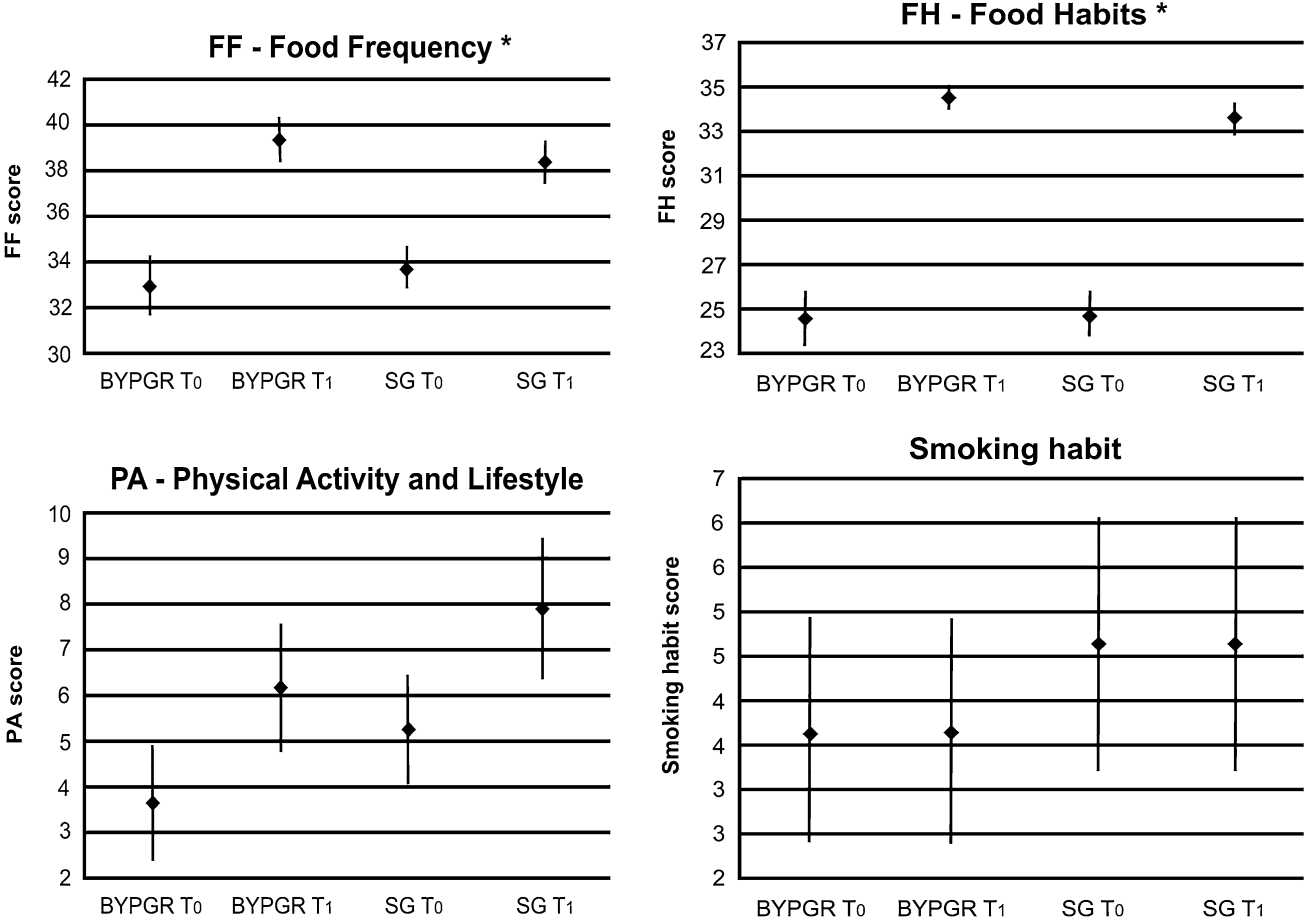
Age and sex adjusted estimates of questionnaire scores. Age and sex adjusted estimates of questionnaire scores (FF, FH and PA) and smoking habits. T0: baseline; T1: after 6 months post bariatric procedures. *Vertical bars* represent 95 % CI. FF, FH and PA scores were placed into tertiles; the worst evaluation was assigned to the lowest tertile while the best evaluation was assigned to the highest. Smoking habits were investigated with six semi-quantitative questions. Each question included multiple choices coded from a score ranging from 1 to 3; the minimum score was assigned to the healthiest habit. * Significance was settled a *p* < 0.05

No statistically significant difference in PA score was detected although both RYGB and SG patients showed a greater PA- section score at T_1_ than at T_0_ (Fig. 1).

As for FF-section, mixed models by surgery reported that PA-section score was not correlated with age, gender, weight, or BMI, either in RYGB patients or in SG patients.

### Results of smoking habit (SH) at T_1_

After their bariatric surgery, neither RYGB nor SG patients changed their smoking habits, which were positively correlated with age (β = 0.15; *p* = 0.002) and female gender (β = 3.8, *p* = 0.004) in SG patients.

Where questionnaire scores did not differ significantly by surgery group, a pooled analysis on the overall sample was performed, but results did not change substantially.

## Discussion

Our study sought to evaluate dietary habits, physical activity and lifestyle, and smoking habits of patients with moderate to severe obesity that have undergone RYGB and SG by means of a self-administered questionnaire. In our patient cohort, we determined that while there was a significant positive change in food frequency and dietary habits in both surgical cohorts, there was no significant change in physical activity and smoking habits after either RYGB or SG.

### Food frequency section (FF) and dietary habit section (FH)

Data on Food Frequency section showed that about 80 % of our bariatric population had Food Frequency scores within the first and the second tertiles, underlying the excess of energy intake and unbalanced diet which is common in patients with obesity (Forestieri 2015). Even if mean FF score did not differ significantly between RYGB and SG subgroups at baseline, the percentage distribution of subjects according to Scores’ Tertiles showed that RYGB patients scored the worse. Indeed, the percentage of RYGB subjects scoring within the 1st tertile was higher than the SG patients.

Since all the patients at T0 were already aware of the bariatric procedure they would undergo, we hypothesize that RYGB patients were more prone to have high expectations regarding weight loss associated with their surgery regardless of their diet, while the SG may have developed a greater awareness of the efforts to be made for diet and lifestyle changes. Another explanation could be that patients with inappropriate dietary behaviors and higher rates of metabolic derangements might have undergone a RYGB as recommended by their surgeon rather than a SG for better long and short term weight and metabolic changes.

After bariatric procedures, FF-score improved significantly both in SG and in RYGB patients (Forestieri 2015). Indeed, considering separately the two surgical procedures, the FF score increased more in RYGB patients than in SG patients, even if not statistically significant. This may be due to RYGB patients having lower scores at baseline and therefore more room for improvement, as well as to changes caused by the surgical techniques per se: RYGB is both a restrictive and malabsorptive procedure and affects mainly weight loss by altering the physiology of weight regulation (Chen et al. 2013) while SG restricts gastric capacity and alters the neuroendocrine hormones involved in satiety and hunger (e.g., ghrelin signaling) (Mans et al. 2015).

Pre-operative data on Dietary Habit (FH) section reported that about 75 % of our bariatric population scored within the first and the second tertiles. This result concurs with results from a prior study, which described unbalanced food consumption and insufficient intake of essential nutrients in preoperative bariatric patients (Correia Horvath et al. 2014). This issue cannot be neglected since studies of bariatric surgery patients presenting for surgery have found significant poor eating habits and nutrients deficiencies that may be exacerbated post surgery (Flancbaum et al. 2006; Ernst et al. 2009).

RYGB patients had the worst FH-score. Additionally, FH-section score was significantly and inversely related with weight at baseline, only in RYGB patients.

After bariatric procedures, FH-score was significantly increased both in RYGB and in SG patients underlying an improvement in dietary habits. This change may be secondary to the nutritional counseling in post-operative course, which reinforces nutrition guidelines. The higher FH-score gain in RYGB patients compared to SG patients could be due to a lower baseline score in RYGB patients.

Finally, mixed models analysis by surgery reported that no correlation between FF-score, BMI, or body weight was highlighted in either the RYGB or in SG patients; however, FH-score was correlated significantly and inversely with weight status both in RYGB and in SG patients. We note that much of the focus in our nutrition program is on the diet quality of the patients after they have undergone weight loss surgery instead of the frequency with which foods are consumed. Often, our bariatric surgery patients may eat more frequent, smaller meals in the postoperative period as they are unable to consume the same volume of food in one sitting compared to preoperatively. This may explain the improved FH scores as opposed to the FF score.

### Physical activity and lifestyle section (PA)

In our study, about 70 % of the patients showed a PA score equally distributed between the 1st and the 2nd tertiles which concurs with the work of Bond et al. (2012) While the two study populations were well-matched for weight and BMI, the number of RYGB patients scoring within the 1st tertile was twice that of SG patients. The PA score was significantly higher in SG than RYGB patients. As RYGB patients were more sedentary at baseline, they needed more pre-operative lifestyle counseling as physical activity is crucial to support weight maintenance in the early and late post-operative course.

In patients with severe obesity, the extra load of weight might represent a severe limitation *per se* for performing regular physical activity, therefore, the overall energy expenditure is reduced and the body weight tends to increase over the long-term (Gradaschi et al. 2014).

Likewise, our results suggest that the sedentary lifestyle of our patients with obesity could not be accounted for only by the physical limitations due to the extra load of body mass. Indeed, the PA score, even if improved after bariatric treatment, did not reach the significance and did not correlate with BMI in either surgical group (RYGB or SG), underlying that the weight reduction did not correspond to a significant improvement in physical activity levels.

Emphasis on the importance of physical activity may not be as pronounced as it should. These patients should be recommended to work directly with a health professional whose primary focus is physical activity.

These results are in line with what has been reported in a comprehensive review on the topic that highlight the challenges faced by patients in adopting a habitual PA program and the assistance that they require to identify and apply appropriate strategies for adhering to PA goals (Wendy and Dale 2013).

### Smoking habits

In our study at baseline, the percentages of current, former, or patients who never smoked both in RYGB and in SG were substantially similar and about one-third of study population were current smokers. However, in contrast with Chatkin et al. (2010), the smoking habits score was not significantly related with BMI or body weight. Interestingly, we report a significant correlation between sex and smoking habits score in SG patients, although female patients showed a higher score than males. The independent effect of preoperative cigarette smoking on bariatric surgical outcomes remains unclear. However, despite the paucity of data, many bariatric surgeons recommend smoking cessation prior to the planned bariatric procedure (Haskins et al. 2014), although it is not considered an absolute contraindication to bariatric surgery.

In contrast with previous findings (Lent et al. 2013; Conason et al. 2013), smoking habits score did not change after surgery in either bariatric procedure and current smokers did not quit smoking. A conceivable lack of awareness of the potential harmful effects of smoking on the perioperative morbidity and on the treatment outcome may partly explain this phenomenon, but this may also be secondary to caregiver’s inattention and lack of dedication to providing patients with the tools necessary to quit smoking.

### Study limitations

This observational study followed the lifestyle and eating habits of subjects with moderate to severe obesity undergoing bariatric surgery over 6 months after bariatric surgery presents some limitations.

The study design is a single-center; further research on adequate sample size from multiple clinical sites is needed.

The evaluation method of food consumption we used, previously validated in adolescents, has not been validated for adults with obesity. Although the results have been positive, it should be recognized as a study limitation.

Besides a possible limitation of any self-administered dietary questionnaires, particularly in the population of patients with severe obesity, is that food intake may be underestimated as well as underreported; we were unable to account for this aspect in our analysis.

Although the study results cannot be generalized, the study results are useful because they allow us to extrapolate about the relationship between food habits and lifestyle modifications in post bariatric patients cared for by a multidisciplinary team.

In summary, the weight loss of our bariatric sample was due primarily to the bariatric procedures, but the positive eating habit modifications we detected is a positive feature and definitely should be considered a positive outcome factor. These results may be in part due to the nutrition counseling the patients received in the postoperative period and indicate the importance of persistent nutritional and medical care of these patients.

Dietary intake and eating behavior after bariatric surgery can affect not only the trend of weight loss but also the patient’s nutritional status which directly affects health. The lack of positive eating habit and dietary modifications are threats to long-term postoperative success (Sarwer et al. 2011).

Unfortunately, the patients did not change their physical activity levels and smoking habits.

Likely, the period of our observation was too short to detect a real change, especially in physical activity level. In addition, the lack of exercise physiology or physiotherapy input pre and postoperatively might have contributed to our patient’s low level of physical activity. Health care professionals should make more efforts to help patients become aware of the significant behavior changes that must be taken in the post-surgical course to achieve weight maintenance over time.

While bariatric surgeries are a stimulus to promote change, these procedures must be coupled with proper information about necessary lifestyle changes to achieve the highest likelihood of long term success (Vartanian and Fardouly 2014). Therefore, long term multidisciplinary care after bariatric surgery is likely to help patients achieve more weight loss and decrease the likelihood of significant weight regain in the post operative course.

## Conclusion

Bariatric surgery remains an important tool to treat patients with moderate to severe obesity who have failed non-surgical approaches to weight management. In order to maximize the response to surgery, patients should be encouraged to adhere to proper dietary habits, physical activity, and stop smoking. Our study shows that patients are likely to improve their food frequency and dietary habits, but their physical activity and smoking behaviors remained unchanged. In order to achieve the highest likelihood of success, patients should work with a multidisciplinary team including a physician, registered dietitian, mental health care professional, and exercise physiologist prior to and after bariatric surgery.
